# Antenatal Diagnosis of Parapagus Conjoined Twins: 3D Virtual and 3D Physical Models

**DOI:** 10.1055/s-0041-1739297

**Published:** 2021-12-21

**Authors:** Pedro Castro, Heron Werner, Ana Paula Matos, Gerson Ribeiro, Jorge Lopes, Edward Araujo

**Affiliations:** 1 Department of Fetal Medicine, Clínica de Diagnóstico por Imagem, Rio de Janeiro, RJ, Brazil; 2 Department of Arts and Design, Pontifícia Universidade Católica, Rio de Janeiro, RJ, Brazil; 3 Department of Obstetrics, Escola Paulista de Medicina, Universidade Federal de São Paulo, São Paulo, SP, Brazil; 4 Medical course, Universidade Municipal de São Caetano do Sul, Campus Bela Vista, São Paulo, SP, Brazil

**Keywords:** conjoined twins, magnetic resonance imaging, three-dimensional models, gêmeos unidos, ressonância magnética, modelos tridimensionais

## Abstract

Conjoined twins (CTs) are a rare complication from monochorionic and monoamniotic twin pregnancies. We describe the use of 3D technologies, including 3D virtual and 3D physical models on prenatal evaluation of one parapagus CT. A 16-year-old G1P0 woman was referred for fetal magnetic resonance imaging (MRI) anatomical evaluation of a CT at 28 weeks of gestation. 3D images of the fetal surface were generated by the software during the examination for spatial comprehension of the relationship between the fetal parts. The pair of CTs died at the 32 ^nd^ week of gestation, a few hours after cesarean section. 3D technologies are an important tool for parental counseling and preparation of the multidisciplinary care team for delivery and neonatal assistance and possible surgical planning for postnatal separation in CTs cases.

## Resumo

Gêmeos unidos (GUs) são uma complicação rara de gestações gemelares monocoriônicas e monoamnióticas. Descrevemos o uso de tecnologias 3D, incluindo modelos 3D virtuais e físicos, na avaliação pré-natal de GU parapagus. Mulher de 16 anos, G1P0, foi encaminhada para avaliação anatômica por ressonância magnética (RM) fetal de GU com 28 semanas de gestação. Imagens 3D da superfície fetal foram geradas pelo software durante o exame para compreensão espacial da relação entre as partes fetais. O par de GUs morreu na 32ª semana de gestação, poucas horas após a cesariana. As tecnologias 3D são uma importante ferramenta de aconselhamento dos pais e de preparação da equipe multiprofissional para o parto e a assistência neonatal, além de possível planejamento cirúrgico para separação pós-natal em casos de GUs.

## Introduction

Conjoined twins (CTs) are a rare complication from monochorionic and monoamniotic twin pregnancies with an incidence ranging from 1:50,000 to 1:100,000 live births. 1 Conjoined twin pregnancies are surrounded by great commotion and prompt disposition of multidisciplinary groups for the care and reception of the pair during pregnancy, delivery, and postnatal life. However, the singularity of the anatomy of CTs can increase the risk of fetal and neonatal demise. Of CTs deliveries, 46% are live born, 26% are stillborn, and 27% are terminated. 2 The exact number of fetal demises on the first half of pregnancy is unknown. This high rate of mortality is extended to the postnatal period: the common presence of cardiac malformations and new circumstances that this singular anatomy is exposed to after birth increases the risk of death of the CTs. This also increases the risk of neonatal demise of one of the CT, requiring urgent surgical separation. 3 Recognizing anatomical singularities is crucial in the prenatal assessment and postnatal assistance of the CTs. For this, prenatal and postnatal imaging studies are fundamental. In the past decades, 3D virtual and 3D physical models (3DPMs) have been described as emerging technologies for the assistance of CTs and parental counseling. The present study aimed at describing the antenatal use of 3D imaging technologies in the assessment of one pair of CTs.

## Case Report

**Fig. 1:**
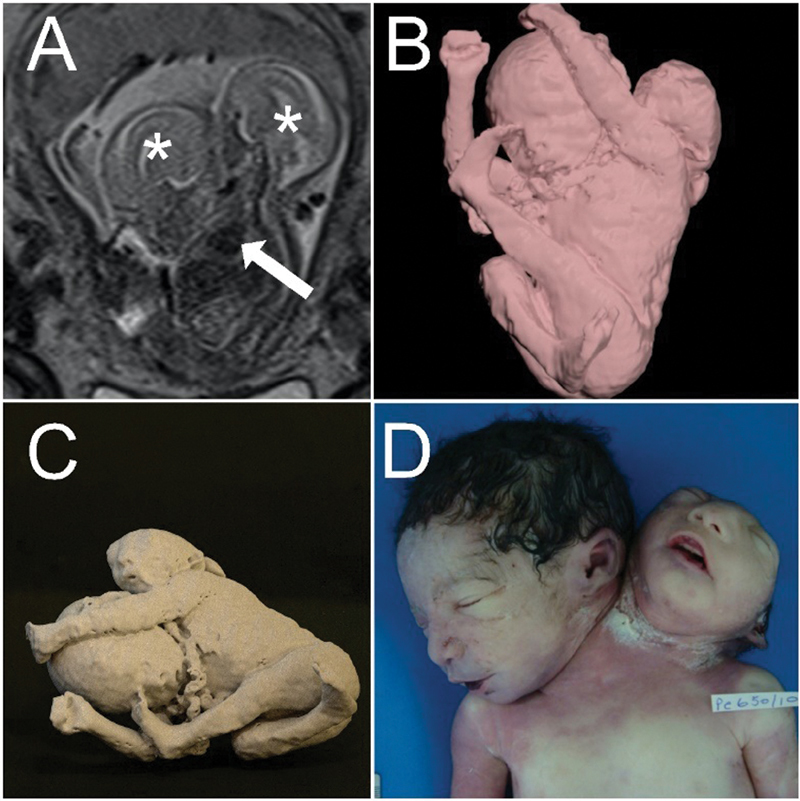
Parapagus, at 28 weeks of gestation. They share the thorax and abdomen and had two spines, and one of the fetuses presented an important encephalocele. ( **A** ) Magnetic resonance imaging was performed during the pregnancy. This image was used for the 3D virtual reconstruction ( **B** ) and 3D physical model printing ( **C** ) for parental counseling. ( **D** ) Postnatal image of the fetuses.

A 16-year-old G1P0 woman was referred for fetal magnetic resonance imaging (MRI) anatomical evaluation of CTs at 28 weeks of gestation. Fetal magnetic resonance imaging (MRI) demonstrated a dicephalic parapagus CT. The thorax was fused, and the heart, aorta, and liver were shared. However, there were two vertebral spines, both fused in the lumbosacral region. One fetus had a massive occipital encephalocele, distorting the cranium. This type of CT was considered lethal, because of one single shared heart and aorta associated with central nervous system malformations. The abdomen presented a cystic mass, hypointense at the T2-weighted sequence and isointense at the T1-weighted sequence. Isotropic images were also obtained. 3D images of the fetal surface were generated by the software (of the MRI machine) during the examination for spatial comprehension of the relationship between the fetal parts. Digital Imaging and Communications in Medicine (DICOM) images were exported for posterior processing using the software 3D Slicer v4.11.10 (Birmingham, United Kingdom). Fetal images were generated for parental counseling and neonatal care programming. Standard Triangle Language (STL) files were generated, and a 3D Physical model (3DPM) was generated (Ultimaker 3 Extended; Utrecht, Netherlands; printing material: Polylactic Acid (PLA); support: Polyvinyl Alcohol [PVA]). At the 32 ^nd^ week, a premature rupture of membranes anticipated the delivery. A cesarean section was performed, and the pair died after a few hours due to cardiopulmonary complications ( Figure 1 ). The neonatology team was informed of the lethal condition and only ventilatory support was offered.

## Discussion

Conjoined twins are a rare occurrence, with an incidence ranging from 1/2,800 in India to 1/200,000 live births in the USA. 4 The anatomical classification is based on the site of fusion, and a standardized nomenclature was suggested by Spencer in 1996. 5 The site of fusion may be associated to the mortality rate. Thoracopagus has a mortality rate of 80%, due to the presence of severe cardiopulmonary abnormalities, while omphalopagus may have a mortality rate of 20%. 6 The anatomical presentation of the CTs is extremely variable, and each CT has a singular anatomy. This singular anatomy leads to the association of multiple major malformations, which are present in more than one-quarter of cases and contribute to the high neonatal mortality rate: > 60% will not survive after birth. 6 Of these, deaths occur on the 1 ^st^ 24 hours in 68% of the cases and within 48 hours in 88% of the cases. 7 To aid in the care of CTs, imaging studies are fundamental, before birth, for parental counseling, individualized prenatal care, and delivery preparation of the multidisciplinary specialized care and, postnatally, for clinical evaluation of the anatomy in cases in which separation is not indicated and in case of surgical separation to provide accurate information for the parents and multidisciplinary care. Recently, many technological advances are being applied and customized to improve the comprehension of the singular anatomy of CTs, including 3D imaging, virtual reality, and 3DPM printing. 8 The formation of a 3D image is the first step after imaging acquisition. The presence of multiple contrasts allows the proper segmentation of the anatomy to be studied. The complex anatomy of CTs requires the presence of a multidisciplinary care team to orient the segmentation of the computer-aided 3D volumes, which can be used for the design of skin and soft tissue flaps, and to calculate the amount of volume necessary in the tissue expander for proper closure of the area of attachment of the CTs. 9 The 3DPM was established during the 1980s, when the patent of a stereolithographic apparatus was registered. The poor results of imaging and computer processing delayed the progress of the technology until the current century. The first description of CT using 3DPM was reported in 2000, when magnetic resonance images of a thoracopagus CT were used for prenatal planning and to provide information to the multidisciplinary care specialist after birth, when surgical separation was immediately required. 10 The 3DPMs are useful for surgical guidance, providing spatial relationship between tissues and organs, and may provide anatomical details that are not visible in 2D images. 11 Vascular connection identification is one of the key factors for the success of the surgical separation of craniopagus CTs. There are many cases reporting the use of 3DPMs in the postnatal care of craniopagus CTs. 3DPMs can provide the physical characteristics and in real scale the relationship between the vessels, the skull, and the brain. 12 From the surgical planning of craniotomy, cranial defects are reconstructed, cranial grafts were designed, and the use of tissue expander and its placement for posterior cranial coverage were programmed. 13 The postprocessing of the stereolithographic files and material of good quality are the determinant factors for a high-quality 3DPM. 14 In this case, the 3D models helped with the parental comprehension of the fetal malformation, with a spatial perception that cannot be obtained by traditional 2D ultrasound. For the neonatology team, the 3D reconstruction helped the neonatal assistance, including the ventilatory support. 3D models can also help on the comprehension of the difficulties (anatomy of organs and vessels) of a possible fetal separation by the pediatric surgery team.

## Conclusion

In conclusion, 3D technologies can be useful in the prenatal evaluation of CTs and are an important tool for parental counseling and possible postnatal surgical separation planning.
